# Mechanisms Underlying the Anti-Inflammatory Activity of Bergamot Essential Oil and Its Antinociceptive Effects †

**DOI:** 10.3390/plants9060704

**Published:** 2020-06-01

**Authors:** Giovanni Enrico Lombardo, Santa Cirmi, Laura Musumeci, Simona Pergolizzi, Alessandro Maugeri, Caterina Russo, Carmen Mannucci, Gioacchino Calapai, Michele Navarra

**Affiliations:** 1 Department of Chemical, Biological, Pharmaceutical and Environmental Sciences, University of Messina, 98168 Messina, Italy; gelombardo@unime.it (G.E.L.); scirmi@unime.it (S.C.); lauramusumeci93@gmail.com (L.M.); simona.pergolizzi@unime.it (S.P.); amaugeri@unime.it (A.M.); cate.russo.22@gmail.com (C.R.); 2 Fondazione “Prof. Antonio Imbesi”, 98168 Messina, Italy; 3 Department of Biomedical and Dental Sciences and Morphofunctional Imaging, University of Messina, 98100 Messina, Italy; cmannucci@unime.it (C.M.); gcalapai@unime.it (G.C.)

**Keywords:** *Citrus bergamia*, bergamot, essential oil, inflammation, pain, carrageenan, paw edema, writhing test, hot plate test

## Abstract

Renewed interest in natural products as potential source of drugs led us to investigate on both the anti-inflammatory and anti-nociceptive activity of *Citrus bergamia* Risso et Poiteau (bergamot) essential oil (BEO). Carrageenan-induced paw edema in rats was used as an experimental model of inflammation. Because of the toxicity of furocoumarins, we performed our study by using the BEO fraction deprived of these compounds (BEO-FF). Treatment with BEO-FF led to a significant inhibition of paw edema induced by a sub-plantar injection of carrageenan. Moreover, histological examination of BEO-FF-treated rat paw biopsies showed a reduction of pathological changes typical of edema. Pre-treatment with BEO-FF significantly reduced interleukin (IL)-1β, IL-6, and tumor necrosis factor (TNF)-α levels in the paw homogenates, as well as nitrite/nitrate and prostaglandin E_2_ (PGE_2_) content in exudates. In addition, BEO-FF possesses antioxidant properties, as determined by cell-free assays. Furthermore, results of the writhing test showed that BEO-FF elicited a pronounced analgesic response, as demonstrated by a significant inhibition of constrictions in mice receiving acetic acid, with respect to control animals, whereas the results of the hot plate test suggested that the supra-spinal analgesia participates in the anti-nociceptive effect of BEO-FF. Our study indicates that BEO-FF exerts anti-inflammatory and anti-nociceptive effects, and suggests its potential role as an anti-edemigen and analgesic drug.

## 1. Introduction

*Citrus* fruits are typically consumed during winter in the Mediterranean diet and, together with their derivatives, have been extensively studied for their health properties, such as anti-inflammatory, antimicrobial, anti-aging, neuroprotective, and anticancer effects [1,2,3,4,5,6,7,8,9,10]. Amongst *Citrus* fruits, *Citrus bergamia* Risso et Poiteau (bergamot), a small tree belonging to the family of Rutaceae, has stimulated an ever-growing interest of the scientific community. This plant is typical of the southern coasts of the Calabria region (Italy), where grows spontaneously. To date, 95% of worldwide bergamot fruit production occurs in the Ionian coast of Reggio Calabria (Calabria, Italy), where a habitat particularly suitable for its cultivation exists. The remaining 5% is crop grown in Greece, Morocco, Iran, Ivory Coast, Argentina, and Brazil. It has been suggested that *Citrus bergamia* is a hybrid between *Citrus aurantium* L. (sour orange) and *Citrus limon* L. Burm.f. (lemon), or a mutation of the latter. Other researchers believe bergamot is a hybrid between *Citrus aurantium* L. (sour orange) and *Citrus aurantiifolia* (Christm. & Panzer) Swingle (lime). Its botanical and geographical origins are still uncertain, although it is present in the Mediterranean area from centuries. It may originate from Calabria region, deriving from a mutation of other species, or it may have been imported by Christopher Columbus. Bergamot fruits are mainly exploited for the extraction of bergamot essential oil (BEO), whereas the juice (BJ), considered formerly a by-product, has recently been appreciated for its relevant biological activities. Indeed, different *in vitro* and *in vivo* studies have demonstrated its antimicrobial [11] and anticancer [12,13] properties, which seem to be due to the flavonoids it contains, since the flavonoid-rich fraction of BJ shows antioxidant and anti-inflammatory capabilities [14,15,16,17,18] and also reduces cancer formation [19]. BEO is broadly used by fragrance industries and in aromatherapy [20]. Moreover, it is exploited for its antimicrobial properties [21], as well as studied for its anticancer [22,23] and cardioprotective [24] effects.

BEO is extracted from the peel of the fruit by a cold-pressing procedure or steam distillation. It comprises a volatile fraction (93–96% of total), consisting in limonene, linalool, linalyl acetate, α-pinene, β-pinene, and γ-terpinen, as well as a non-volatile fraction (4–7%), whose main components are represented by coumarins and psoralens, such as bergapten, bergamottin, and citropten [25]. These compounds (coumarins and furocoumarins) are reported to have a broad spectrum of biological activities, including antimicrobial [26], anti-platelet aggregation [27], anti-mutagenic [28], and anti-inflammatory [29,30]. The non-volatile fraction of BEO inhibits oxidative stress occurring in arteries injured by balloon angioplasty and reduces both lectin-like oxidized low-density lipoprotein receptor-1 (LOX-1) expression and neointima proliferation [24]. The volatile fraction is essentially constituted by monoterpenes, whose biological effects are well-known [31,32].

Inflammation is a tangled protective response against biological, chemical or physical injuries, caused by signaling molecules generated by leukocytes, macrophages, and mast cells, as well as by the activation of complement factors, which lead to edema formation as a result of extravasation at the inflammatory site. It can be distinguished as either acute or chronic, depending on the type of stimulus and the effectiveness of the inflammatory process resolution. Acute inflammation begins quickly and persists for a few hours or a few days, whereas, if the inflammatory response fails to remove its cause, a chronic phase occurs. Currently, the main therapeutic approach to fight inflammation is represented by the use of anti-inflammatory drugs, which are accompanied by a number of side effects. This has resulted in the search for new therapeutic strategies, often represented by natural products. In this context, essential oils (EOs), aromatic plant secondary metabolites, are increasingly gaining attention as anti-inflammatory agents [33,34,35].

On these bases, the aim of the present study was to investigate the mechanisms underlying the anti-inflammatory effects of BEO in paw edema induced by carrageenan (CAR), as well as to evaluate its anti-nociceptive effect. Because furocoumarins are well known for their toxicity, we carried out this study using a BEO deprived of furocoumarins (BEO-FF).

## 2. Results

### 2.1. BEO-FF Reduced the Edema Formation in CAR-Injected Hind Paw of Rats

To assess the anti-inflammatory activity of BEO-FF, we employed the CAR-induced paw edema model, an established model widely used to investigate acute inflammation *in vivo*. As shown in Table 1, CAR-induced paw edema reached a maximum level after 4 h from CAR injection, decreasing the volume during the subsequent hours.

The effects of BEO-FF on the edematogenic response induced by CAR in rat paw are shown in Table 1. The phytocomplex was administered intraperitoneally (i.p.) 1 h before CAR, and the paw volumes were measured at the following times: 0 (baseline), 1, 2, 3, 4, and 5 h after CAR injection. Compared with the vehicle group (seed oil), we observed a significant reduction in paw volumes in the animals pre-treated with either 86.2 mg/kg or 431.2 mg/kg of BEO-FF (100 and 500 µL, respectively) before CAR injection (*p* < 0.01). The reduction of paw edema by BEO-FF was already observed at both doses starting from the first hour after the CAR administration, reaching the greatest anti-edemigen effect 3 h later (51% and 59.7%, respectively). The dose of 8.6 mg/kg of BEO-FF did not suppress paw edema compared with the vehicle group (data not shown), while 4 mg/kg indomethacin (IND), a well-known anti-inflammatory drug, significantly suppressed it (65%; Table 1). Altogether, these results indicate that BEO-FF exerts anti-edemigen activity in tissues affected by acute inflammation.

Since both doses (86.2 and 431.2 mg/kg) of phytocomplex exerted a similar anti-edemigen effect, further experiments were carried out, employing only the lowest dose.

### 2.2. Effects of BEO-FF on the Release of Pro-Inflammatory Cytokines Evoked by CAR

**Figure 1:**
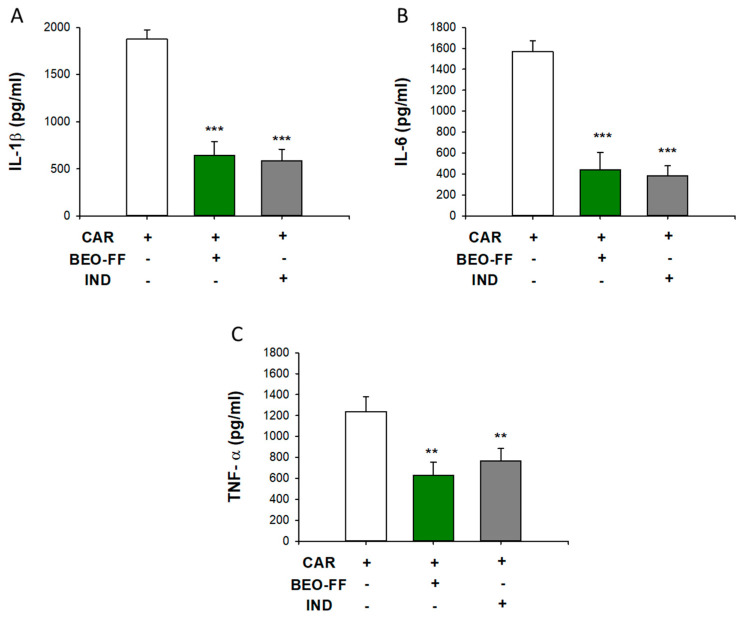
**Effects of BEO-FF on the release of pro-inflammatory cytokines induced by CAR.** Rats were treated with 86.2 mg/kg (100 μL) of BEO-FF 1 h before the intraplantar injection of CAR, and, after 5 h, the subcutaneous tissue of hind paw was removed and homogenized. Levels of interleukin (IL)-1β (**A**), IL-6 (**B**), and tumor necrosis factor (TNF)-α (**C**) in the paw homogenates were determined by ELISA. Data are presented as mean ± SEM of the values found in five animals per group. ** *p* < 0.01 and *** *p* < 0.001 vs. CAR group.

In order to investigate the mechanism(s) of action of BEO-FF, some mediators of early phase inflammation were taken in account. Five hours after CAR injection, we sacrificed the animals and the subcutaneous tissue of hind paw was removed and homogenized. The levels of pro-inflammatory cytokines interleukin (IL)-1β, IL-6, and tumor necrosis factor (TNF)-α increased after CAR injection and are shown in Figure 1. The pre-treatment with 86.2 mg/kg of BEO-FF significantly prevented the rise of IL-1β, IL-6, and TNF-α levels evoked by CAR (−65%, −72%, and −50% vs. CAR group, respectively; *** *p* < 0.001) with effectiveness comparable to that of IND (** *p* < 0.01). BEO-FF alone did not exert any effect on the levels of cytokines examined in this study.

### 2.3. BEO-FF Counteracted the CAR-Induced Release of PGE_2_ and Nitrate/nitrite in the Rat Paw Exudates 

**Figure 2:**
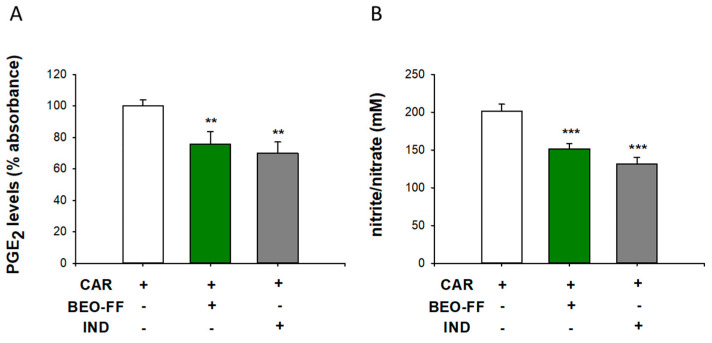
**BEO-FF reduced PGE_2_ and nitrite/nitrate levels in the CAR-induced exudates in rat paw.** PGE_2_ (**A**) and nitrite/nitrate (**B**) levels in paw exudates from BEO-FF-treated or -untreated CAR-injected animals were measured by ELISA assay or Griess reaction, respectively. Data are mean ± SEM of five animals for each group. * *p* < 0.05 vs. CAR group.

As shown in Figure 2, the systemic treatment (i.p.) with BEO-FF 86.2 mg/kg 1 h before the CAR injection led to a significant reduction of prostaglandin E_2_ (PGE_2_) levels (≈30%) and NO production in paw exudates (* *p* < 0.05 vs. CAR group), as observed through ELISA assay and Griess reaction, respectively. These results are comparable with those of the IND group.

### 2.4. BEO-FF Ameliorated CAR-Induced Histological Damage

**Figure 3:**
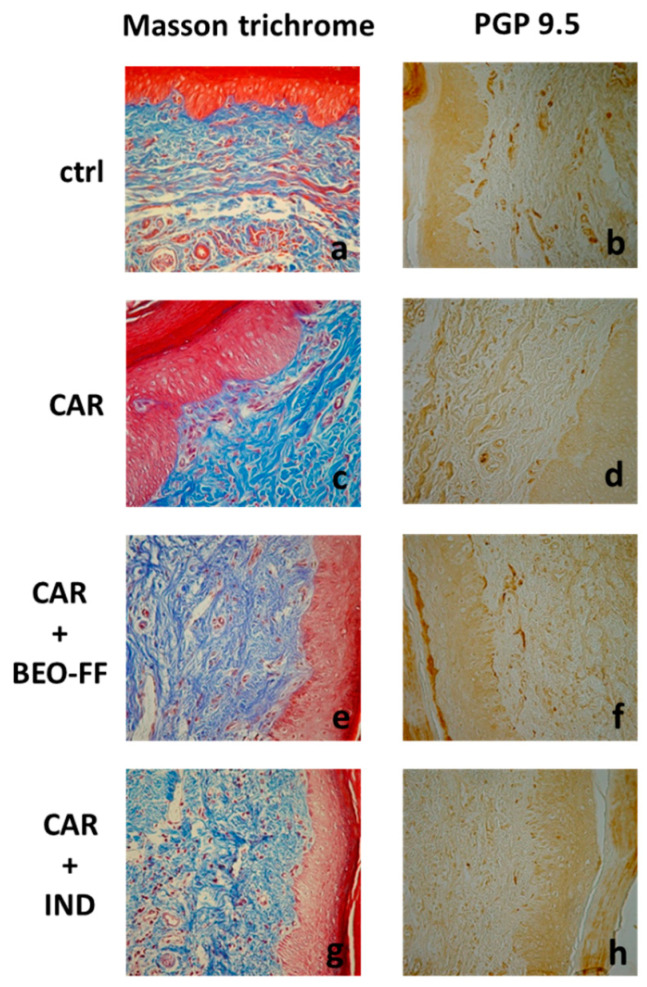
**BEO-FF ameliorated the pathological features typical of edema.** Five hours after CAR injection, rats were sacrificed, the hind paws were removed, and their sections were examined by optical microscopy. Representative photos of paw specimens from animals belonging to control (**a**,**b**), CAR-inflamed (**c**,**d**), pre-treated with BEO-FF (**e**,**f**), or indomethacin (IND) (**g**,**h**) groups stained with Masson trichrome or a rabbit polyclonal antibody against PGP 9.5. Original magnification: ×40.

Rat hind paws were collected 5 h after CAR injection and examined by optical microscopy. Representative paw sections illustrated tissue from the various experimental groups stained with both Masson trichrome and a rabbit polyclonal antibody against protein gene product (PGP) 9.5 (Figure 3). The microscopic observation of rat paws injected with CAR showed the typical histopathological features of an inflamed tissue (Figure 3c). The paw sections stained with Masson trichrome showed alterations of the epidermal layers, in which an appreciable modification of the cellular structure with numerous vacuolated cells was observed. In the superficial dermis, an increase of the size and number of dilated blood vessels that cause a profound decomposition of collagen fibers was shown. The immunohistochemical analysis performed with an antibody against PGP 9.5 showed the reduction of the nerve network (Figure 3d). Figure 3a shows paw tissues of rats that were not injected with CAR, in which a normal skin pattern was observed. The immunohistochemical analysis showed the normal pattern of fibers with the typical trend in the superficial dermis immediately below the basal membrane (Figure 3b). Figure 3e shows tissues of rats treated with BEO-FF (86.2 mg/kg) and then injected with CAR. In this case, compared to the sections of paw from CAR group, we observed a marked improvement in the edema, with a reduction in the number of vacuoled cells in the epidermis, a decrease in the thickness of the collagen fibers, and a reduction in the vascular network in the superficial dermis. Moreover, there was a clear reduction in the number of infiltrating elements. The immunohistochemical analysis (Figure 3f) showed nervous fibers to be well represented, as well as their regular progression to the basal membranes, thus conferring an aspect similar to that observed in paw sections from control group (Figure 3b). The photo present in Figure 3g corresponds to tissue section of rats treated with IND and then with CAR. As expected, there was a trend to normalize the edema frame. The immunohistochemical analysis highlighted a modest expression of nervous elements (Figure 3h).

### 2.5. Antioxidant Capacity of BEO-FF in Cell-Free Models

**Figure 4:**
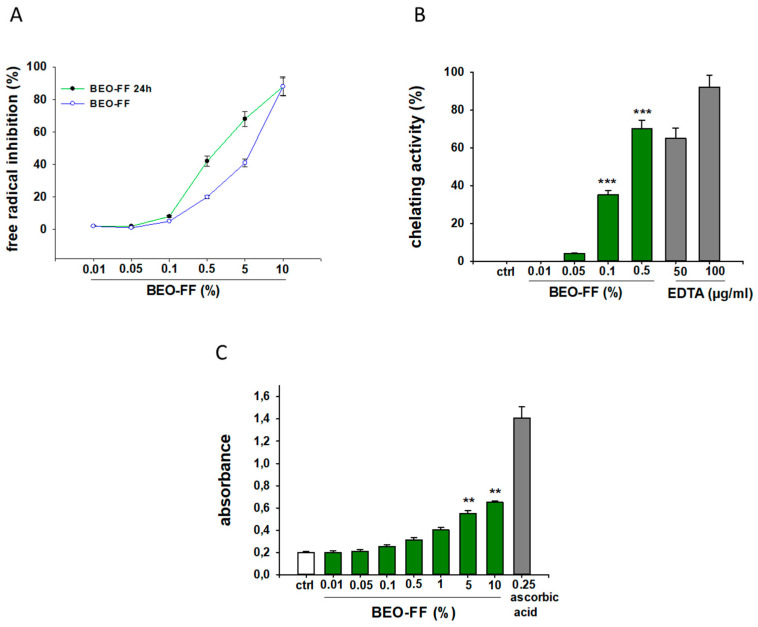
**Antioxidant activity of BEO-FF.** (**A**) 2,2-Diphenylpicrylhydrazyl (DPPH) free-radical scavenging test; (**B**) chelating activity assay; (**C**) ferric reducing power assay. Data are the mean ± SEM of three experiments performed in triplicate. * *p* < 0.05, ** *p* < 0.01, and *** *p* < 0.001 vs. controls.

It is well known that the majority of natural products with anti-inflammatory properties also exert anti-oxidant effects. In order to examine this issue, we evaluated the antioxidant and radical scavenging properties of BEO-FF through three abiotic tests. Figure 4 shows that BEO-FF possesses scavenging properties, as assessed by the 2,2-diphenylpicrylhydrazyl (DPPH) test (A); capability to reduce ferric ion, as evaluated by the reducing power test (B); and ability to chelate pro-oxidant metal, as determined by the chelating activity assays (C), suggesting its role as both a primary and secondary anti-oxidant drug. 

### 2.6. BEO-FF Exerts a Anti-Nociceptive Effect

Often, the anti-inflammatory properties of natural products are associated to analgesic activity, which is commonly linked to their capability to fight phlogosis, whereas sometimes they act in the central nervous system. In order to evaluate the anti-nociceptive effects of BEO-FF and to distinguish between peripheral and central analgesic effects, we carried out both the writhing and hot plate tests in mice. 

**Figure 5:**
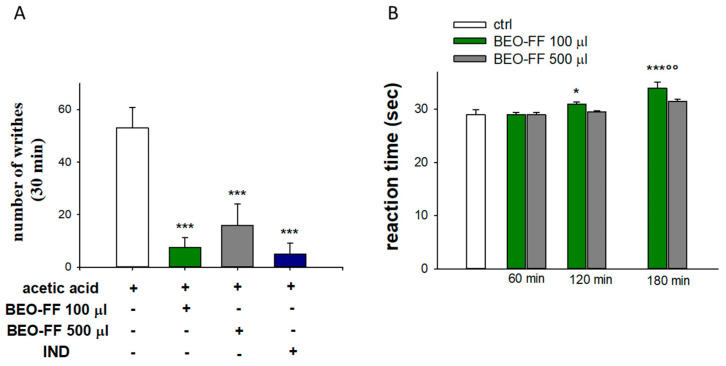
**Analgesic effect of BEO-FF in mice.** (**A**) Peripheral anti-nociceptive activity of BEO-FF (86.2 or 431.2 mg/kg) evaluated by the writhing test, showing the significant inhibition of constrictions by BEO-FF in mice receiving acetic acid (1.2%). Data are expressed as number ± SEM of writhes. ** *p* < 0.01 and *** *p* < 0.001 vs. control. (**B**) BEO-FF (86.2 mg/kg) increased the time of reaction in the hot-plate test. Results are expressed as mean ± SEM of latency time (second). * *p* < 0.05 and *** *p* < 0.001 vs. control; °° *p* < 0.01 vs. 120 min.

The results of the writhing test are shown in Figure 5A. Compared to animals receiving vehicle, BEO-FF led to a pronounced analgesic response, as demonstrated by a significant inhibition of abdominal constrictions due to the acetic acid. Indeed, BEO-FF at the doses of 86.2 or 431.2 mg/kg reduced the number of acetic acid-induced abdominal writhes by 86% and 70%, respectively (*p* < 0.001 and *p* < 0.01 vs. vehicle). The analgesic effect of the lowest dose of BEO-FF was superimposable to those exerted by IND, supporting its anti-inflammatory effect and hence suggesting its role as peripheral analgesic drug.

BEO-FF administered at the dose of 431.2 mg/kg 1 h prior to subjecting mice to the hot plate test did not produce any significant effect compared to the 86.2 mg/kg dose (Figure 5B). On the contrary, treatment with the lowest dose (86.2 mg/kg) elicited a pronounced analgesic response at central level, as demonstrated by the significant increase in the reaction time at the second and third hour after BEO-FF treatment (*p* < 0.05 for the second hour and *p* <0.001 at the third hour vs. control; *p* < 0.05 vs. 120 min; Figure 5B).

## 3. Discussion

Nowadays, medicinal plants are extensively used in folk medicine for the treatment of diverse inflammatory conditions [36]. In the continuous search for novel and safer natural products to constrain inflammation, essential oils (EOs) have gained the attention of both researchers and users all over the world. EOs are characterized by a strong odor, are rarely colored, and generally have a lower density than that of water. They are a liquid mixture of volatile and non-volatile substances, extracted from both aromatic and medicinal plants through steam- or hydro-distillation or Soxhlet. In some cases, such as with EOs from *Citrus* fruits, the process takes place by a suitable mechanical process without heating. EOs have a complex composition, mainly characterized by the presence of terpenes (oxygenated or not) but mostly monoterpenes and sesquiterpenes, to which antioxidant, anti-inflammatory, and anti-infective properties are attributed [31,32,37,38,39]. In nature, EOs are secondary metabolites that are synthetized to protect plants against pathogens such as bacteria, viruses, and fungi; to prevent from being attacked by insect pests; as well as to attract or repel insects, which is the case when EOs are present in pollen and seeds. Commercially, they have a wide range of applications in the fragrance and cosmetic industries, as well as in food and pharmaceutical industries. EO use in human health is linked to their biological properties, which have numerous applications [20,21,34,35,36,40,41,42,43]. Those of *Citrus* EOs have been recently reviewed [5,33], among which the anti-inflammatory action of BEO has been already reported [44], albeit without investigating the mechanism. 

In our study, the anti-inflammatory effect of BEO-FF was evaluated in CAR-induced paw edema in rats, a suitable experimental model of acute inflammation [45]. Development of edema in rat paw is a biphasic event. The initial phase is ascribed to the release of histamine, serotonin, and bradykinin, while the second phase of edema is attributed to the release of proteases, prostaglandins, and lysosomes [46]. Herein, we present data showing the significant reduction of paw edema by BEO-FF systemically administered (i.p.) 1 h before CAR injection, which we studied in the first phase of inflammation. According to Annamalai and co-workers [46], in our experiment, edema was peaked 3–4 h post-injection of CAR in hind paw, although the highest anti-edemigen effect of BEO-FF was observed 3 h after the pro-inflammatory injury. The anti-inflammatory effect of BEO-FF was also confirmed by histological and immunohistochemical analysis, carried out in sections of hind paw tissue collected 5 h after CAR injection, showing that BEO-FF is able to reduce the pathological features typical of edema.

It is known that the levels of some pro-inflammatory cytokines, such as IL-1β, IL-6, and TNF-α, increase during CAR-induced edema, playing a pivotal role in the onset and perpetuation of the inflammatory process [46], thus representing important targets for developing new anti-inflammatory drugs. In line with other research demonstrating the ability of EOs to modulate these pro-inflammatory cytokines [34,47,48], in our study, BEO-FF reduced the levels of up-mentioned cytokines in the paw homogenates, thereby suggesting a possible mechanism underlying BEO-FF anti-inflammatory activity. In addition, NO and PGE_2_, generated by nitric oxide synthases (NOS) and cyclooxygenase (COX), respectively, are key pro-inflammatory mediators that are widely implicated in inflammatory diseases [49], hence the inhibition of NO and PGE_2_ release being one of the main mechanisms through which EOs exert their anti-inflammatory activity [50]. Therefore, measuring PGE_2_ and nitrite/nitrate levels in hind paw exudates, we found that BEO-FF reduced the amount of both PGE_2_ and nitrite/nitrate. The latter could have been related to the antioxidant properties of BEO-FF, as we demonstrated by different cell-free assays.

Several studies have shown that EOs and their constituents possess analgesic activity, which is frequently related to their anti-inflammatory properties [31,51,52,53,54]. The acetic acid-induced writhing test has been widely used as a screening tool in order to assess the analgesic properties of several substances [52,55]. Acetic acid is used to trigger irritative stimulus in the peritoneum, which in turn induces abdominal constrictions. They are caused by the peripheral sensitization of nociceptors through the release of nociceptive endogenous mediators, including prostaglandins and cytokines such as TNF-α, IL-1β, and IL-8 [56]. The BEO-FF reduced the number of abdominal contortions at both tested doses. Since acetic acid is responsible for promoting the release of bradykinin, prostaglandins, serotonin, histamine, cytokines, and noradrenaline, among other nociceptive mediators, the aforementioned effect may be due to the inhibition of the release of these nociceptive mediators. Therefore, we suggest that the anti-inflammatory effect of BEO-FF plays a crucial role in the peripheral analgesia we witnessed, undoubtedly along with other mechanisms such as the interaction with the opioid system, as previously claimed by Sakurada and co-workers [57], in relation to the whole BEO, linalool, and linalyl acetate. We also reported that BEO-FF increased the latency time, as evaluated by the hot plate test, employed to evaluate central analgesic drugs [58], thereby indicating that the supra-spinal analgesia participates in the antinociceptive effect of BEO-FF. As reviewed by Guimarães and co-workers [59], the analgesic mechanism of monoterpenes, representing the main components of BEO-FF, is multiple, thus suggesting that, overall, BEO-FF acts as a multi-target analgesic drug.

It is known that monoterpenes, among which linalool, limonene, and linalyl acetate, representing the major constituents of BEO-FF, possess anti-inflammatory and analgesic capability [31,51,58,60,61]. Therefore, at least in part, the anti-inflammatory and analgesic effect of BEO-FF could be attributable to these compounds, although, in this study, we did not investigate which monoterpenes were responsible for these outcomes. On the other hand, we and others believe that a phytocomplex, consisting in a pool of substances, is able to induce a specific biological effect by interacting simultaneously with a wide plethora of targets. Therefore, its pharmacological effect is attributable to the combined action of the single molecules that compose it. Interestingly, in our experiments, BEO-FF had superimposable effectiveness to that of IND, a recognized anti-inflammatory drug used in therapy.

## 4. Conclusions

This study supplies pharmacological evidence regarding the anti-inflammatory effect of BEO-FF, showing the mechanisms underlying this activity. In addition, our results provide new insight on its anti-nociceptive activity, suggesting its potential as an analgesic drug. 

## 5. Materials and Methods

### 5.1. Drug

In this study, we used BEO-FF because of the well-known toxicity of furocoumarins. Its chemical composition has been previously described by Costa and co-workers [25]. Nevertheless, prior the beginning of this study, we performed a new HPLC analysis, whose results reflect the qualitative/quantitative composition of BEO-FF already reported. The main components are limonene, linalyl acetate, and linalool (Table 2). The drug was stored at 4 °C and dissolved in seed oil to the desired concentration just prior to use.

### 5.2. Animals

Adult male Wistar rats (180–200 g) and Swiss mice (20–25 g) (Harlan, Milan, Italy) were used to study the anti-inflammatory or anti-nociceptive effects, respectively. The animals were kept in standardized conditions (temperature 22 ± 2 °C; humidity 60% ± 4%, natural light), in groups of five per standard cage with water and food ad libitum. They were acclimatized in the animal facility for at least 1 week before testing. Animal care was in compliance with Italian legislation on protection of animals used for experimental and other scientific purposes (D. M. 116/92), as well as with the Europe regulations (O. J. of E. C. L 358/1 12/18/1986). All efforts were made to minimize animal suffering and to use only the number of animals necessary to produce reliable results. 

In the experiments aimed to assess the anti-inflammatory effect of BEO-FF, we divided rats randomly into five groups of five animals each:

(1) Rats who received only the vehicle (seed oil) i.p.;
(2) Rats treated with 8.6 mg/kg of BEO-FF i.p.;
(3) Rats treated with 86.2 mg/kg of BEO-FF i.p;
(4) Rats treated with 431.2 mg/kg of BEO-FF i.p.;
(5) Rats treated with 4 mg/kg of IND i.p.

In the experiments designed to evaluate the anti-nociceptive effect of BEO-FF, we randomly allocated mice into four groups of five animals each:

(1) Mice who received only the vehicle (seed oil) i.p.;
(2) Mice treated with 86.2 mg/kg of BEO-FF i.p;
(3) Mice treated with 431.2 mg/kg of BEO-FF i.p.;
(4) Mice treated with 4 mg/kg of IND i.p.

### 5.3. CAR-Induced Paw Edema in Rats

All rats received CAR, as described below.

Anti-inflammatory activity was evaluated on the basis of inhibition of CAR-induced hind paw edema [45]. Edema was induced in the hind paw by intraplantar injection of 1% carrageenan diluted in physiological saline (100 µL/paw). Paw volume (mL) was measured with a plethysmometer (Ugo-Basile, Varese, Italy) immediately prior to the injection of CAR (T_0_) and thereafter at hourly intervals for 5 h. BEO-FF was administered 1 h before CAR injection. Results are presented as the difference in volume between those detected at baseline (T_0_) and those assessed 1, 2, 3, 4, and 5 h after CAR injection. After the final assessment, animals were sacrificed, paws were removed, exudate was collected, and the tissue was homogenated.

### 5.4. Measurement of IL-1 β, IL-6, and TNFα

The hind paw tissue was homogenized in phosphate buffer saline (PBS) solution containing 0.05% Tween 20, 0.1 mM phenylmethanesulfonyl fluoride (PMSF), 0.1 mM benzamethonium chloride, 10 mM ethylenediaminetetraacetic acid (EDTA), 2 µg/mL aprotinin A, and 0.5% bovine serum albumin (BSA), centrifuged at 3000× *g* for 10 min and stored at −80 °C until analysis. The levels of cytokines were evaluated by using standard sandwich ELISA technique, according to the manufacturer’s recommendations (R&D Systems, Minneapolis, MN, USA).

### 5.5. Determination of PGE_2_ Levels in the Rat Paw

Exudate from paws of BEO-FF-treated and -untreated animals injected with CAR was collected and then stored at −80 °C until required for PGE_2_ assay. Levels of PGE_2_ were measured using an ELISA technique using a PGE_2_ assay kit (Cayman Chemical Company, Ann Arbor, MI, USA), according to the manufacturer’s specifications. 

### 5.6. Quantification of Nitrite/Nitrate Concentrations

Paws were gently centrifuged at 250× *g* for 20 min to recover the edematous fluid that was stored at −80 °C. Nitrite (NO_2_^−^) and nitrate (NO_3_^−^) levels were measured by the Griess reaction. Briefly, aliquots (100 μL) of exudate were diluted (1/10) with ultrapure water and incubated at room temperature with 250 μL substrate buffer (0.1 M imidazole, 210 μM nicotinamide adenine dinucleotide phosphate (NADPH), 3.8 μM flavine adenine dinucleotide, pH 7.6) in the presence of nitrate-reductase (*Aspergillus niger*, 10 UI/l; Sigma-Aldrich, Milan, Italy) for 45 min to convert NO_3_^−^ to NO_2_^−^. Total NO_2_^−^ (NO_2_^−^ + NO_3_^−^) was analyzed by reacting the samples with Griess reagent (58 mM sulfanilamide and 3.8 mM naphtalene-ethylene diamine dihydrochloride in 0.5 M H_3_PO_4_). The absorbance was measured at 540 nm (iMark microplate absorbance reader, Bio-Rad Laboratory, Milan, Italy). Amounts of NO_2_^−^ were estimated by a linear standard curve for NaNO_2_ between 1 and 150 μmol/L.

### 5.7. Histological and Immunohistochemical Examination

Sub-plantar tissues were fixed in 4% paraformaldehyde in phosphate buffer 0.2 M, pH 7.4, at 4 °C for 4 h. Then, samples were dehydrated in graded ethanol (30°–100°) and embedded in Bioplast (Bioptica, Milan, Italy). Rotating microtome sections (5 μm thickness) were deprived of paraffin with xylene and stained with Masson trichrome. All samples were observed and photographed by an AxiosKop microscope (Carl Zeiss, Milan, Italy). Immunohistochemical studies were performed as follows: section of 5 μm thickness were incubated with a rabbit polyclonal antibody against PGP 9.5 (dilution 1:500, AbD Serotec, Bio-Rad Laboratory) for 12 h in a humid chamber at 4 °C, and then incubated for 1 h with a goat anti-rabbit immunoglobulin G (IgG) in goat-peroxidase conjugated (dilution 1:100, Sigma-Adrich, Milan, Italy). Peroxidase activity was visualized by incubating the sections in a solution of 0.015% 3-3′-diaminobenzidine in 0.01 M Tris buffer, pH 7.6, containing 0.005% H_2_O_2_.

### 5.8. Antioxidant Activity of BEO-FF

The antioxidant capability of BEO-FF was assessed by cell-free assays such as the DPPH free-radical-scavenging test, the ferric reducing power, and the chelating activity assay.

The ability of BEO-FF to scavenge DPPH^•^ free radicals was measured as reported [62]. The DPPH solution (7.6 × 10^−5^ M) in dichloromethane was prepared fresh daily prior to UV measurements. Various volumes (10, 20, 50, 80, 100, or 200 µL) of the BEO-FF stock solution (1 g/mL) dissolved in dichloromethane were removed and placed into the vials to give the following concentrations: 10, 20, 50, 80, 100, or 200 mg/mL (corresponding to 0.01%, 0.05%, 0.1%, 0.5%, 1%, and 5%). The DPPH solution (1 mL) was added to each of these vials. After sample solutions were left to stand for 30 min in the dark (25 °C), we measured the absorbance at 517 nm using a Prixma UV-VIS spectrophotometer. Butylated hydroxytoluene (BHT) was used as reference. Blank samples containing the same amount of dichloromethane and DPPH solution were also prepared and measured. In its free radical form, DPPH^•^ has an absorption band at 517 nm, which disappears upon reduction by an anti-radical compound. Therefore, the BEO-FF scavenging activity was measured as the decrease in absorbance of samples versus DPPH standard solution. The results are expressed as percentage inhibition (mean values ± standard error of the mean; SEM) of the DPPH.

The reducing power test was performed according to the method of Ferlazzo et al. [63]. A total of 200 µL of BEO-FF at different concentrations (from 0.01% to 10%) was mixed with 0.5 mL of phosphate buffer (200 mM, pH 6.6) and 0.5 mL of potassium ferric cyanide (1%), and incubated at 50 °C for 20 min. Thereafter, 0.5 mL of trichloroacetic acid (10%) was added to the reaction mixture, and centrifuged for 10 min at 3000 rpm. The upper layer of solution (0.5 mL) was mixed with 0.5 mL of distilled water and 0.1 mL of FeCl_3_ (0.1%), and absorbance was measured at 700 nm. The blank solution was prepared as above, but contained distilled water instead of BEO-FF. A solution of ascorbic acid 0.25 mM (Sigma-Aldrich) was used as a reference. The results are expressed as mean value ± SEM of absorbance. An increased absorbance of the reaction mixture indicated increased reducing power.

The chelating activity of BEO-FF toward ferrous ions was determined as described by Dinis et al. [64] with modifications. Briefly, 100 μL of 2 mM FeCl_2_ was added to 1 mL of different concentrations of BEO-FF methanolic solution (0.01%, 0.05%, 0.1%, 0.5%, and 1%). After 5 min, the reaction was initiated by the addition of 100 µL of 5 mM ferrozine solution. The mixture was vigorously shaken and left to stand at room temperature for 10 min. The absorbance of the solution was thereafter measured at 562 nm. A reaction mixture containing 100 μL methanol instead of substance solution served as a control. EDTA (50 and 100 mg/mL) was used as the chelating standard. BEO-FF chelating activity (mean value ± SEM) is expressed as the percentage inhibition of ferrozine–Fe^2+^ complex formation.

The antioxidant assays were carried out in triplicate and repeated three times.

### 5.9. Antinociceptive Activity

The ability of the BEO-FF to reduce pain was evaluated in mice by both the hot plate and the writhing tests following Peana and co-workers [51]. In the acetic acid-induced writhing response assay, mice were injected i.p. with 1.2% acetic acid solution in normal saline in a volume of 5 mL/kg body weight, 30 min after i.p. injection of IND (4 mg/kg) or BEO-FF 86, 24, or 431.2 mg/kg. Animals of the control group received acetic acid and saline solution. Immediately after the injection of acetic acid, we isolated each mouse in an individual observation chamber (25 × 20 × 25 cm). Antinociception was recorded by counting the number of writhes after the algic compound injection for a period of 10 min. A writhe was indicated by abdominal constriction and full extension of hind limb. Results are expressed as percentage ± SEM. of writhe inhibition compared with that of controls. The antinociceptive activity of BEO-FF was also studied by the hot plate test. Pain reflexes in response to a thermal stimulus were measured using a Hot Plate Analgesia Meter (Ugo Basile, Italy). The surface of the hot plate was heated to a constant temperature of 55 ± 0.5 °C. Mice were placed on the hot plate (25.4 × 25.4 cm) surrounded by a clear acrylic cage (19 cm tall, open top), and the start/stop button on the timer was activated. The time between placement of the animal on the hot plate and the occurrence of either the licking of the fore or hind paws, shaking, or jumping off the surface was recorded as response latency. First, we measured the basal latency time before the administration of drug or saline solution. Mice with baseline latencies of >60 s were eliminated from the study. Then, hot plate latencies were re-determined at 60, 120, and 180 min after BEO-FF or IND administration. Results were expressed as mean ± SEM of latency time (second).

### 5.10. Statistical Analysis

Data show mean ± SEM with the number of observations indicated in parenthesis. Statistical analysis was either performed by one-way ANOVA followed by post-hoc Tukey test or, where appropriate, by Student’s *t*-test.

## Figures and Tables

**Table 1:** Effects of bergamot essential oil (BEO) deprived of furocoumarins (BEO-FF) in carrageenan (CAR)-induced hind paw edema in rats.

Drug	Dose (mg/kg)	Mean Change in Paw Edema (%)					3 h Edema Inhibition (%)
		1 h	2 h	3 h	4 h	5 h	
**CARRAGEENAN**	1	31.3 ± 0.08	59.4 ± 0.01	84.8 ± 0.009	88.1 ± 0.013	78.7 ± 0.03	-
**BEO-FF (100 µL)**	86.2	10 ± 0.056 **	26.7 ± 0.08 **	41.2 ± 0.01 **	44.3 ± 0.042 **	47.3 ± 0.013 **	51
**BEO-FF (500 µL)**	431.2	15.3 ± 0.05 **	23.8 ± 0.055 **	34.1 ± 0.07 **	43 ± 0.098 **	39.3 ± 0.067 **	59.7
**INDOMETHACIN**	4	9.8 ± 0.04 **	22.6 ± 0.072 **	29.6 ± 0.03 **	30.2 ± 0.065 **	29.1 ± 0.05 **	65.03

Pre-treatment with BEO-FF (86.2 and 431.2 mg/kg) led to a significant inhibition of CAR-induced paw edema starting from 1 h after the CAR injection up to 5 h. The percentage of increase in paw volume of the right hind paws of each rat at each time point was calculated. Data represent the mean ± standard error of the mean (SEM) of five animals per group. ** *p* < 0.01 vs. CAR group.

**Table 2:** Main compounds found in BEO-FF.

Content of Coumarins and Psoralens		Volatile Constituents (%)	
**Citropten**	traces	**ß-pinene**	5.01
**Bergapten**	traces	**limonene**	36.87
**Bergamottin**	traces	**γ-terpinene**	4.67
**5-Geranyloxy-7-methoxycoumarin**	traces	**linalool**	12.24
		**linalyl acetate**	32.12
